# Symptomatic Response After Laparoscopic Cholecystectomy for Symptomatic Gallbladder Polyps: A Patient Questionnaire

**DOI:** 10.7759/cureus.70223

**Published:** 2024-09-26

**Authors:** Ahmed Mahmoud Askar, Bharathi Akula, Aftab Mohammed Arif, John Isherwood, Eyad Issa, Neil Bhardwaj, Ashley Dennison, Giuseppe Garcea

**Affiliations:** 1 Department of Hepatopancreatobiliary Surgery, University Hospitals of Leicester, Leicester, GBR; 2 Department of Hepatopancreatobiliary Surgery, University hospital of Leicester, Leicester, GBR; 3 Department of Hepatopancreatobiliary Surgery, University Hospitals of Leicester NHS Trust, Leicester, GBR

**Keywords:** benign gallbladder diseases, gallbladder polyp, gallbladder ultrasound, laparoscopic cholecystectomy, post-cholecystectomy

## Abstract

Aim

To assess the effect of laparoscopic cholecystectomy (LC) in relieving the biliary type symptoms in patients with gallbladder polyps (GBPs) and to determine the positive and negative predictive values (PPV, NPV) of abdominal ultrasound (US) for the pre-operative detection.

Methods

The data were retrieved from our tertiary hepatobiliopancreatic (HPB) center database for all patients who had an LC as a treatment for symptomatic GBPs between 2013 and 2022. The pre-operative US and postoperative histology reports were reviewed. Patients were contacted and asked to fill in a questionnaire using the Accurx® software (Accurx UK) asking them about the degree of symptom relief following their surgery. Subsequently, the responses were correlated with polyp size, and the data collected was used to determine the PPV and NPV of the US examination for the identification of GBPs.

Results

Seventy patients had GBPs reported on pre-operative US and/or postoperative histology reports. Thirty-six patients (51.4 %) replied to our questionnaire. Twenty-four patients (66.6 %) reported complete relief of pain post-operatively, eight (22.2%) had a significant improvement of symptoms but still had ongoing mild discomfort, two (5.5%) are still experiencing discomfort which has not reduced following their cholecystectomy and two patients (5.5%) were unsure of the degree of improvement. Overall, 89 % of the patients reported a complete or major improvement in their symptoms after LC.

Nine patients with putative GBPs on their pre-operative US had negative final histology while 26 patients whose initial US report showed only gallstones (GSs), had GBPs confirmed by their histology report. The prevalence of GBPs in our snapshot cohort is 21.6%, with a PPV of US of 83.02%, an NPV of 90.37%, and an accuracy for detecting GBPs of 89.16%.

Conclusion

Although LC continues to be the gold standard for the management of symptomatic gallstone disease, assessing the benefit of symptomatic GBPs is presently lacking. This study has demonstrated that the majority of patients with symptomatic GBPs experience a complete resolution or major improvement of their symptoms following surgery. Furthermore, a significant number of patients undergoing surgery for putative GSs will have GBPs demonstrated following histological examination, suggesting that these two conditions either coexist or the pre-operative assessment by US is not sufficiently reliable. Randomized controlled trials are needed to define the cohorts who require surgery or are most likely to benefit.

## Introduction

Gallbladder polyps (GBPs) are elevated lesions of the mucosal surface of the gallbladder [1,2] and the management of GBPs varies from center to center [3,4], for large polyps ≥ 10 mm due to the increased risk of malignancy, surgery is generally the preferred option [5]. Surgery is also frequently recommended for smaller polyps when they are felt to be responsible for the patient’s symptoms and other alternative diagnoses have been excluded [3]. Most guidelines consider polyp size when recommending a management strategy for GBPs [6] but it is important to consider other patient factors such as age and fitness [7], ethnicity [8], and primary sclerosing cholangitis [9]. There is a paucity of available literature examining the benefit of cholecystectomy for the relief of symptoms attributed to GBPs [10]. In this study, we have examined a cohort of patients who underwent laparoscopic cholecystectomy (LC) for symptomatic GBPs and have replied to a questionnaire allowing assessment of the benefit post-operatively in respect of the resolution or improvement of pain and calculation of the positive and negative predictive (PPV, NPV) values of pre-operative US in detecting GBPs.

## Materials and methods

Inclusion criteria

A snapshot of patients who had an LC between 2013 and 2022 was identified from our tertiary hepatopancreatobiliary (HPB) center database. All adults aged ≥ 18 years old who underwent LC for GBPs were included. Despite using different keywords and codes to ascertain identifying the correct group, results included some patients who underwent surgery for gallstone disease (GSD), the inclusion criteria were extended to include this group and were used for comparison.

Data collection

The pre-operative US and postoperative histology reports were reviewed to identify and confirm the indication of surgery and the presence of GBPs and/or GSs. Patients were contacted using Accurx® software [11] with a questionnaire containing three questions assessing the degree of symptom relief after the surgery (Table 3). Questionnaire replies from both groups were compared.

Exclusion criteria

Patients who didn’t reply to the questionnaire were excluded from the evaluation of the primary outcome of this study which is the assessment of the effect of LC in relieving the biliary type symptoms in patients with GBPs but were included in the assessment of other outcomes.

Data analysis

Only simple data analysis was required for this study. Patients’ replies to the questionnaire were analyzed to assess the effect of LC in relieving the biliary type symptoms in patients with GBPs as the main cohort of this study and were compared to the second group where surgery was performed for GSD. We also correlated the size of the reported GBPs with the degree of postoperative symptom relief. Data collected from all included patients was used to assess the PPV and NPV of the US in accurately detecting GBPs.

## Results

**Figure 1:**
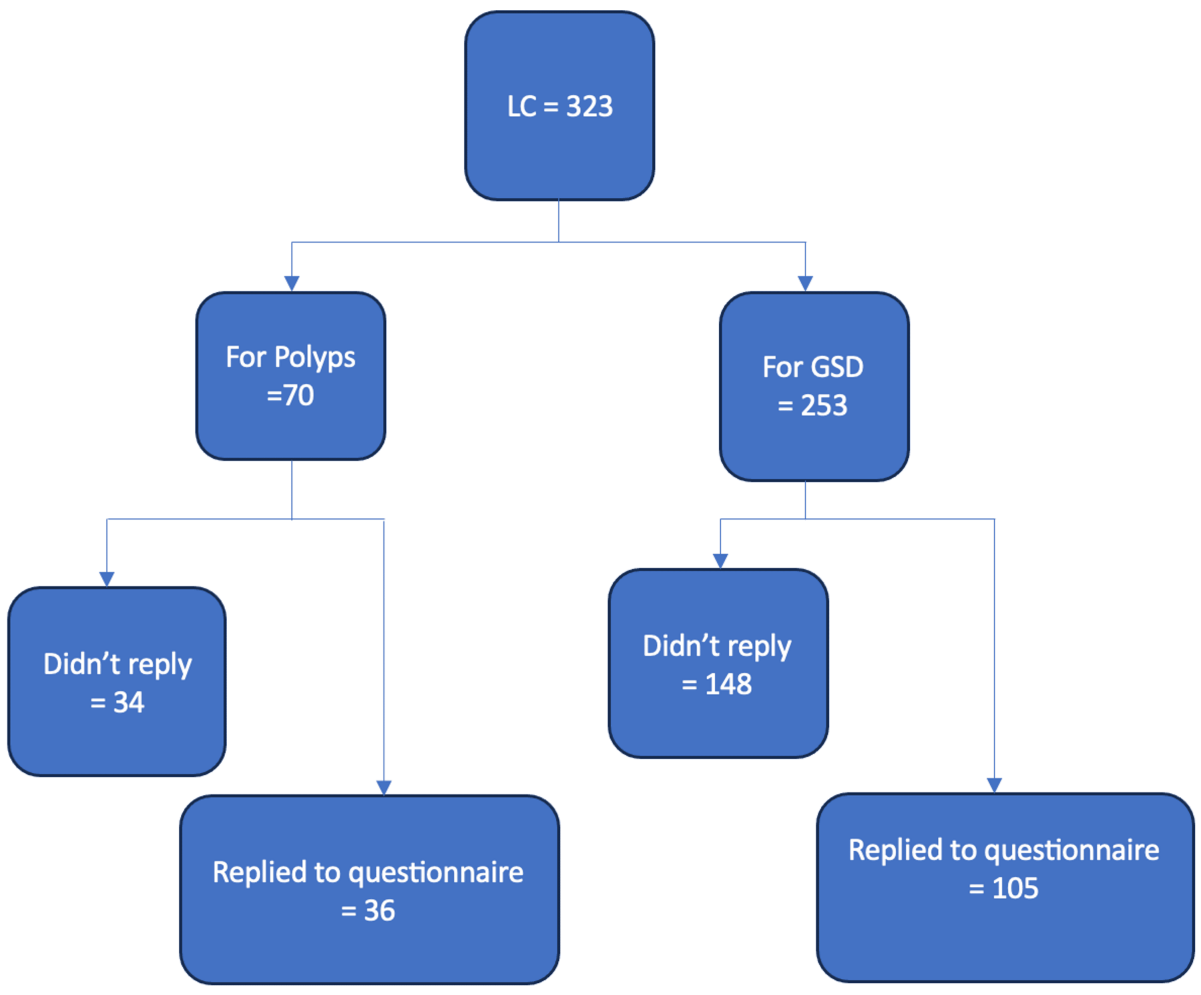
Patients’ inclusion criteria LC: laparoscopic cholecystectomy; GSD: gallstone disease

Three hundred and twenty-three patients who had an LC were identified (Figure 1). Fifty-three patients had a pre-operative US reporting one or more GBPs although only 44 had GBPs confirmed by their final histology. An additional 26 patients were found to have GBPs in their post-operative histology reports despite pre-operative US having not demonstrated them, making the total number of patients included in our cohort 70 (21.6%); 21 males and 49 females, age range 30-84 years with median of 58 years. Thirty-six (51.4 %) of the 70 patients replied to our questionnaire with 24 (66.6%) reporting complete relief of pain post-operatively, eight (22.2%) a significant improvement of symptoms but with ongoing mild discomfort, two (5.5%) still experiencing the same level of pre-operative discomfort while and two (5.5%) patients unsure of the degree of improvement after LC (Table 1).

**Table 1:** All questionnaire replies LC: laparoscopic cholecystectomy; GBPs: gallbladder polyps; GSD: gallstone disease

Patients’ group	Total number replied	Complete relief of symptoms	Ongoing mild discomfort	Severe discomfort	Not sure
LC GBPs	36	24 (66.6%)	8 (22.2%)	2 (5.5%)	2 (5.5%)
LC for GSD	105	70 (66.6 %)	25 (23.8 %)	7 (6.6 %)	3 (2.8%)
Total	141	93 (65.9%)	33 (23.4 %)	9 (6.3%)	5 (3.5%)

LC was performed for presumed GSD in 253 patients, 76 males, and 177 females, the age range of 16-88 years with a median of 60 years. A hundred and five (41.5%) replied to our questionnaire. Seventy (66.6%) patients reported complete relief of pain postoperatively, 25 (23.8 %) had a significant improvement of symptoms but with ongoing mild discomfort, seven (6.6%) were still experiencing the same level of pre-operative discomfort, and three (2.8%) were unsure of the degree of improvement after LC.

In total, 323 patients underwent LC as a treatment for symptomatic GSs and/or GBPs, 97 males and 244 females with an age range of 16-88 years with a median age of 59 years. One hundred and forty-one (43.6%) replied to our questionnaire and 93 (65.9%) patients reported complete relief of pain post-operatively, 33 (23.4%) had a significant improvement of symptoms but with ongoing mild discomfort, 9 (6.3 %) were still experiencing the same level of pre-operative discomfort while 5 (3.5%) were unsure of the degree of improvement after LC (Table 1).

All patients presented with abdominal pain but frequently with additional symptoms including nausea, vomiting, bloating, and jaundice. The smallest polyp size reported was 2 mm and the largest was 13 mm with a median polyp size of 7 mm. The predominant pathology was cholesterol polyps in 55/70 (78.6%), with the remaining types described as adenomyomatosis, adenomyomas, adenomyosis, adenomatous hyperplasia, and granulation tissue polyps. No malignant polyps were diagnosed in our cohort.

There was no clear correlation between polyp size and symptom improvement after LC. Those who reported a continuation of the same level of pre-operative pain or were not sure whether symptoms had improved postoperatively had polyps of between 7 and 9 mm or polyp size was not reported in either the US or the histology reports. Patients who reported complete resolution or significant improvement of pain had polyp sizes ranging between 2 and 13 mm (Table 2).

**Table 2:** The correlation between symptoms and polyp size

Degree of symptom relief	Gender	Age	Number of polyps	Size of largest polyp (mm)
Complete relief	F	59	Multiple	Not available
	F	71	Multiple	3
	F	53	2	10
	F	62	Multiple	12
	F	35	1	Not available
	F	70	1	8
	M	61	Multiple	Not available
	F	54	1	7
	F	35	Multiple	Not available
	F	54	Multiple	Not available
	M	76	Multiple	Not available
	F	72	Multiple	10
	F	80	Multiple	Not available
	F	70	Multiple	Not available
	M	52	Multiple	Not available
	F	56	Multiple	Not available
	F	75	1	2
	M	50	1	13
	M	67	1	12
	M	37	6	6
	F	50	1	5
	M	58	Multiple	7
	F	35	Multiple	Not available
Mild discomfort	F	37	Multiple	Not available
	F	38	1	7
	F	78	Multiple	Not available
	F	32	1	5
	F	55	Multiple	Not available
	F	46	Multiple	2
	F	50	1	7
	F	58	Multiple	3
Same level of pre-operative discomfort	F	56	3	7 mm
	F	63	Multiple	Not available
Not sure	F	65	1	9 mm
	F	70	Multiple	Not available

PPV and NPV of US in our cohort

Nine patients with putative GBPs on their pre-operative US had a negative final histology while 26 patients whose initial US report showed GSs with no polyps had GBPs on their final histology report. The prevalence of GBPs in our snapshot cohort was 21.6% and the PPV and NPV of the US were 83.02% and 90.37%, respectively with an accuracy of US in detecting GBPs of 89.16%.

## Discussion

Cholecystectomy is the gold standard for the management of GSD [12-13] but its role in managing symptomatic GBPs is uncertain [10]. Most available guidelines dealing with GBPs understandably focus on the incidence of malignant transformation in GBPs and related risk factors [5-9] and it is common to recommend surgery to patients with symptomatic GBPs [3]. There remains a paucity of available literature however making it difficult to confidently assess the benefit of cholecystectomy in this group [10]. In a systematic review of 5674 laparoscopic cholecystectomies, Qandeel et al. assessed the outcome in 42 patients (0.7%) who had cholecystectomy for GBPs demonstrating good symptomatic relief in 95% [14]. Our study of 36 patients who had LC for GBPs who responded to the questionnaire shows comparable results with 89 % reporting complete resolution or major improvement in their symptoms after LC.

The size of the GBP has always been considered an important factor when offering surgery to patients due to the associated risk of malignancy [3,5,6]. There are however no presently available studies that correlate the size of the GBPs with a degree of symptom relief post-operatively. In our study, there was no obvious correlation, as some of the patients who reported significant improvements in symptoms had GBPs up to 13 mm while some of those who reported a continuation of symptoms had GBPs as small as 7 mm although the small number of patients and the missing data due to the retrospective nature of the study limits our ability to be dogmatic.

GBPs are a frequent finding in US [4] but it is not uncommon for the subsequent histology reports to refute the imaging or identify polyps that had not been demonstrated. In our study, we found a PPV of 83.02% and NPV of 90.37% with an accuracy of US for the detection of GBPs of 89.16%. Zhang et al. compared conventional US with contrast-enhanced US (CEUS) when assessing 105 gallbladder lesions which were subsequently correlated with the post-operative histology reports. For conventional US, PPV was 60.9%, NPV 96.3 %, and accuracy was 88.6% but this was improved by the use of CEUS to 80%, 98.8%, and 95.2, respectively [15]. Newer techniques such as endoscopic ultrasound (EUS) are also evolving to improve the assessment of GBPs to facilitate better management strategies and enable clinicians to have a more informed discussion with patients [16,17]

Our results do demonstrate the benefit of LC in treating symptomatic GBPs regardless of the size of the polyp. With the present lack of high-quality literature, it is clear that there is an urgent need for randomized control trials to assess the benefit of LC in treating symptomatic GBPs. Careful pre-operative investigations to exclude other causes such as peptic ulcer disease, renal and colonic pathology, and referred pain are essential with the known limitations of conventional US in accurately identifying GBPs.

The main limitation of this study was extracting the data for patients who underwent LC specifically for GBPs. Despite using different keywords to limit results to this group, search results included those who underwent surgery for GSD, the inclusion criteria were extended to include this group and were used for comparison.

## Conclusions

Although LC continues to be the gold standard for the management of symptomatic GSD, assessing the benefit of symptomatic GBPs is presently lacking. This study has demonstrated that the majority of patients with symptomatic GBPs experience a complete resolution or major improvement of their symptoms following surgery. Furthermore, a significant number of patients undergoing surgery for putative GSs will have GBPs demonstrated following histological examination, suggesting that these two conditions either coexist or the pre-operative assessment by US is not sufficiently reliable. Randomized controlled trials are needed to define the cohorts who require surgery or are most likely to benefit.

## Appendices

**Table 3:** Patient questionnaire Dear [Patient Name], We are contacting you because you have had surgery in the past on your gallbladder. If this is the case, please click on the link below to answer some questions so that we can understand more about your recovery. This should only take 2 minutes to complete.

	Question	Yes	No
1	Did you have your gallbladder removed because it was causing you pain and discomfort?	☐ Yes	☐ No
2	Did you experience any other symptoms before you had your gallbladder removed?	☐ Yes	☐ No
3	Have your symptoms improved or gone away since your gallbladder surgery?	☐ Yes	☐ No
